# Prevalence of Frailty Indicators and Association with Socioeconomic Status in Middle-Aged and Older Adults in a Swiss Region with Universal Health Insurance Coverage: A Population-Based Cross-Sectional Study

**DOI:** 10.1155/2014/198603

**Published:** 2014-10-22

**Authors:** Idris Guessous, Jean-Christophe Luthi, Christopher Barrett Bowling, Jean-Marc Theler, Fred Paccaud, Jean-Michel Gaspoz, William McClellan

**Affiliations:** ^1^Unit of Population Epidemiology, Division of Primary Care Medicine, Department of Community Medicine, Primary Care, and Emergency Medicine, Geneva University Hospitals, 4 Rue Gabrielle-Perret-Gentil, 1211 Geneva 14, Switzerland; ^2^Community Prevention Unit, University Institute of Social and Preventive Medicine, 10 Route de la Corniche, 1010 Lausanne, Switzerland; ^3^Department of Epidemiology, Rollins School of Public Health, Emory University, 1518 Clifton Road, Atlanta, GA 30322, USA; ^4^Birmingham/Atlanta Geriatric Research Education and Clinical Center, Atlanta Veterans Affairs Medical Center, 1670 Clairmont Road, Atlanta, GA 30322, USA; ^5^Department of Medicine, Emory University, 1518 Clifton Road, Atlanta, GA 30322, USA

## Abstract

Frailty prevalence in older adults has been reported but is largely unknown in middle-aged adults. We determined the prevalence of frailty indicators among middle-aged and older adults from a general Swiss population characterized by universal health insurance coverage and assessed the determinants of frailty with a special focus on socioeconomic status. Participants aged 50 and more from the population-based 2006–2010 Bus Santé study were included (*N* = 2,930). Four frailty indicators (weakness, shrinking, exhaustion, and low activity) were measured according to standard definitions. Multivariate logistic regressions were used to determine associations. Overall, 63.5%, 28.7%, and 7.8% participants presented no frailty indicators, one frailty indicator, and two or more frailty indicators, respectively. Among middle-aged participants (50–65 years), 75.1%, 22.2%, and 2.7% presented 0, 1, and 2 or more frailty indicators. The number of frailty indicators was positively associated with age, hypertension, and current smoking and negatively associated with male gender, body mass index, waist-to-hip ratio, and serum total cholesterol level. Lower income level but not education was associated with higher number of frailty indicators. Frailty indicators are frequently encountered in both older and middle-aged adults from the Swiss general population. Despite universal health insurance coverage, household income is independently associated with frailty.

## 1. Introduction

Frailty is a biological syndrome that results from cumulative declines across multiple physiologic systems and causes vulnerability to adverse outcomes [1]. Frailty predicts adverse outcomes such as hospitalization and death [2, 3] and is considered as a modifiable predictor of disability [4]. Frailty is partly reversible, especially in its initial phase [4], and the identification of frail adults followed by effective interventions could potentially prevent disability and other adverse outcomes. Studies among older adults, generally defined as people aged 65 years and over, suggest that the prevalence of frailty is variable and has been reported to range from 4.0% to 59.1% of studied populations [5]. Comparable information about the prevalence of frailty among populations younger than 65 years is limited [6]. Gathering data among middle-aged subjects is important given that frailty phenotype finds its ideal application in nondisabled subjects [7].

Socioeconomic status (SES) may contribute to the variability in prevalence among older individuals [8–10]. Life (including early life) course social and health conditions linked to frailty among older adults are often present earlier in adulthood [11, 12]. However the association of SES with frailty among middle-aged adults has not been extensively studied. We address these issues in this study by describing the prevalence of frailty and assessing the association between frailty and lower SES in a population-based study of middle-aged and older adults with uniform access to healthcare.

## 2. Methods

### 2.1. Study Population

We used data from the Bus Santé study. The Bus Santé study is an annual cross-sectional population-based survey that collects information on cardiovascular risk factors in the Canton of Geneva (Switzerland) [13]. Subjects are randomly selected throughout each survey year to generate a representative sample of the canton's noninstitutionalized residents aged 35 years and older. Eligible subjects are identified using a list of legal residents provided by the local government. Stratified random sampling is used to select participants by gender within each 10-year age stratum, selecting the number of participants that is proportional to the corresponding population distribution. The 2006–2010 participation rates varied between 60% and 68%. The Bus Santé study complied with the Declaration of Helsinki and was approved by the Institutional Ethics Committee of the University of Geneva. All participants gave written informed consent.

### 2.2. Data Collection

Participants are examined by trained health professionals. Each participant completes several self-administered, standardized questionnaires. Anthropometric measures, including height, weight, and waist circumference, and blood pressure (BP) are obtained following specified protocols. In a temperature-controlled room, body weight was measured with the subject lightly dressed without shoes using a medical scale (precision 0.5 kg), and standing height is measured using a medical gauge (precision 1 cm). Body mass index (BMI) was defined as weight/height^2^. Waist and hip circumferences (cm) were measured in duplicate with an anthropometric tape while the subjects were wearing light clothing. Waist circumference was measured at the minimum circumference between the iliac crest and the rib cage. Hip circumference was measured at the maximum protuberance of the buttocks, and the waist-to-hip ratio was calculated. BP was measured three times on the right arm after at least 10 min rest in the seated position, using a clinically validated automated oscillometric device (Omron HEM-907, Matsusaka, Japan) with a standard cuff or a large cuff if arm circumference was ≥33 cm. The average of the three BP readings was used for analyses. Hypertension was defined as mean SBP ≥ 140 mmHg or mean DBP ≥ 90 mmHg or presence of antihypertensive medication.

Smoking was defined as present if a participant reported to be a current smoker at the time of examination. Diabetes was defined as a fasting glucose ≥7 mmol/L and/or the presence of antidiabetic drug treatment (insulin or oral drugs). Other cardiovascular diseases (CVDs) and risk factors were defined by affirmative answers to the following questions: “Have you ever been told that you had high cholesterol/myocardial infarction/arterial thrombosis?”

Glucose, total and HDL plasma cholesterol, and triglycerides were assayed by using commercially available enzymatic kits (Bayer Technicon Diagnostics, CV 1.4%, 1.2%, and 1.5%, for glucose, cholesterol, and triglycerides, resp.).

### 2.3. Frailty Indicators

Frailty indicators were assessed by using standardized measures in participants aged 50 or more. These indicators approximated four of the five frailty constructs described by Fried et al. [2]. Frailty indicators used are defined below.

#### 2.3.1. Weakness

Weakness was defined as the gender specific and BMI adjusted lowest 20% grip strength. The grip strength test was performed on the right hand, using the mean of the three measurements with a hydraulic hand dynamometer (Baseline Evaluation Instruments, New York).

#### 2.3.2. Low Activity

Low activity was defined as the gender specific lowest 20% energy expenditure. Energy expenditure in kcal/week was measured using a validated physical activity frequency questionnaire (PAFQ) [14].

#### 2.3.3. Shrinking

Shrinking was defined as self-reported unintentional weight loss. Participants were asked whether they had lost unintentionally weight during the past year: “In the last 12-month* [sic]*, have you unintentionally lost weight?”

#### 2.3.4. Exhaustion

Exhaustion was defined as self-reported exhaustion. Participants were asked whether they had a feeling of generalized weakness in the past year: “In the last 12-month* [sic]*, did you have a feeling of generalized weakness or lack of energy?”

### 2.4. Socioeconomic Status

Self-reported education and income were used to characterize SES. Education level and monthly household income were categorized into 3 groups (education: high (≥13 years), middle (9–12 years), and low (<8 years); monthly household income: <4,999 CHF, 5,000–9499 CHF, and >9,500 CHF (1 CHF ≈ 1 US$ in September 2014)).

### 2.5. Statistical Analyses

To account for the survey year-specific age and gender structure estimates and associations were weighted using the 2010 Geneva Census population. Weighted means (standard errors, SE) and frequencies of study variables were calculated. Participants were grouped into three groups according to the number of frailty indicators: 0 frailty indicators, 1 frailty indicator, and 2 or more frailty indicators. The latter category was used because the number of participants with 3 or 4 frailty indicators was small. To account for the sampling procedure, survey procedures (SAS surveyfreq, surveymeans, and surveyreg) were used with the weights option added for each procedure using the 2010 Geneva Census population age and gender structures as weights. We compared groups using the Rao-Scott Chi-square test and* F* test for categorical and continuous variables, respectively.

We used polytomous multivariate logistic regression to determine and test the associations of potential factors, including socioeconomic factors, CVD, and cardiovascular risk factors, with the number of frailty indicators (1 frailty indicator versus 0, 2+ frailty indicators versus 0). Polytomous logistic regression modeling allows for simultaneous estimation of the probability of multiple outcomes. Middle-aged and older categories were defined as age of 50–64.9 years and ≥65 years, respectively. To determine whether the adjusted associations of SES (i.e., household income and education) with frailty indicators were different in middle-age and older participants, statistical interaction of age category with household income and education was tested, respectively. Citizenship was self-reported and categorized as Swiss or non-Swiss. Analyses were limited to participants with all variables of interest available.

All *P* values were 2-tailed with significance set at <0.05. All analyses were performed using SAS software version 9.2 (SAS Institute, Inc., Cary, North Carolina).

## 3. Results

### 3.1. Participants' Characteristics

Out of the 3,117 subjects aged 50 years and more who participated in the Bus Santé study between 2006 and 2010, 2,930 (95.0%) were included in the analyses. The major reason for exclusion was absence of blood sample. Participants' characteristics are presented in Table 1. Overall mean (SE) age was 60.0 (0.1) and 26.4% were aged 65 years or more. Half were women and 78.8% were Swiss. Hypertension and diabetes were present in 47.5% and 6.6%, respectively. Monthly household income of <4,999 CHF, 5,000–9499 CHF, and >9,500 CHF was reported by 23%, 42%, and 35% of the cohort.

### 3.2. Frailty Indicators

**Figure 1:**
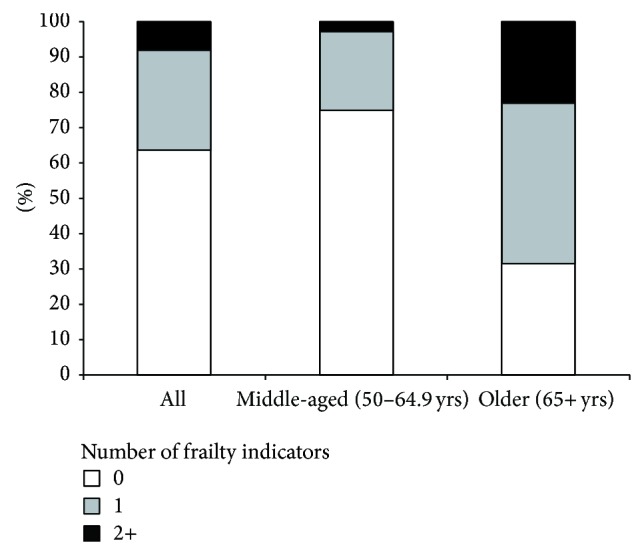
Prevalence of participants with 0, 1, and 2 or more frailty indicators, by age group, Bus Santé study, Geneva, 2006–2010, *N* = 2,930.

Low activity and weakness were the most frequently observed frailty indicators; 18.3% and 13.8% of the participants presented low activity and weakness, respectively. Exhaustion was reported in 7.0% and shrinking in 6.3% (Table 1). Among middle-aged participants exhaustion, shrinking, and low activity were present in 7.6%, 5.7%, and 14.4%, respectively. No middle-aged participant presented weakness. Among participants aged 65 or more, weakness, exhaustion, shrinking, and low activity were present in 52.2%, 5.4%, 7.7%, and 29.0%, respectively. Among the 2,930 participants, 0, 1, or 2 or more frailty indicators were present in 63.5%, 28.7%, and 7.8%, respectively (Table 2). Among participants with 2 or more frailty indicators, 75.3% were of the age of 65 and older. Among middle-aged participants (50–64.9 years), 0, 1, and 2 or more frailty indicators were present in 75.1%, 22.2%, and 2.7%, respectively. Among older participants (≥65 years), 31.2%, 46.6%, and 22.3% had 0, 1, and 2 or more frailty indicators (Figure 1).

Characteristics differed across group defined by the number of frailty indicators present (Table 2). The mean age and proportion of women and Swiss increased with the increasing number of frailty indicators. Both education level and monthly household income were associated with the number of frailty indicators.

### 3.3. Determinants of the Number of Frailty Indictors

In the polytomous multivariate logistic regression analyses, the number of frailty indicators was positively associated with age, hypertension, and current smoking status and negatively associated with male gender, BMI, waist-to-hip ratio, and total cholesterol (Table 3). The associations with male gender and BMI were significant only when comparing participants with 1 frailty indicator to participants without frailty indicator. The association with total cholesterol was significant only when comparing participants with 2 or more frailty indicators to participants without frailty indicator.

Monthly household income was negatively and independently associated with having more frailty indicators. Compared to participants with high monthly income level, participants with low monthly income level were 31% more likely to have 1 frailty indicator and two times more likely to have 2 or more frailty indicators. Education level was not independently associated with the number of frailty indicators.

We found a statistical interaction of age category (50–64.9 versus ≥65 years) with household income but not with education. The positive associations of household income with 2 or more frailty indicators were stronger among middle-aged participants than older participants.

## 4. Discussion

Using population-based data from a Swiss region with universal health insurance coverage, we found that the prevalence of frailty indicators was high in adults aged 50 or more. Frailty indicators were frequently reported in middle-aged adults (50–64.9 years) too; one-fourth had at least one frailty indicator.

The prevalence of frailty indicators increased with age which is in line with previous observations. Previous population-based studies have however rarely reported the prevalence of frailty among large enough sample of middle-aged participants. The prevalence of frailty in community-dwelling middle-aged and older Europeans participating in a large survey in 2004 has been reported; it ranged from 5.8% to 27.3% [6]. Compared to other countries, Switzerland was characterized by a low prevalence of frailty. Of note, only 882 participants were included in the Swiss part of the 2004 survey and the participation rate was low (37.6%). Out of the 882, only 470 participants were of the age of 50–65 yrs. Among this middle-aged group, the prevalence of exhaustion, weakness, shrinking, and low activity was 25.4%, 2.5%, 5.2%, and 11.2%, respectively [6]. Thirty-six percent and 1.3% were prefrail (1-2 frailty indicators) and frail (3+ frailty indicators), respectively [6]. Using a larger sample in an urban region of Switzerland (Geneva), our results further suggest that frailty is not uncommon in middle-aged adults.

Frail subjects are characterized by rapid decline of functional status even after minor perturbations and effective interventions to prevent disability in frail older persons who have been identified [15]. The identification of frail middle-aged adults followed by effective interventions could potentially prevent disability in older persons. Of note, given the general lack of data on frailty among middle-aged adults, there is currently no robust information on the impact of frailty in middle-aged participants on health in later life. However, given that frailty is a continuous process and that the risk of adverse outcome is sought to increase gradually from nonfrailty to prefrailty and frailty [2], it is likely that—without intervention—frailty before age of 65 years increased the risk of frailty and adverse outcome thereof after age of 65 years. Nevertheless, the natural history of frailty during middle and late life course remains to be described in cohort studies and the efficacy of interventions in middle-aged frail adults determined by experimental studies.

We found a negative association between monthly household income and the number of frailty indicators. Compared to adults with monthly household income <9,500 CHF, adults with monthly household income <5,000 CHF were more likely to have frailty indicators. This relationship persisted after adjustment for major potential confounders including age, gender, nationality, smoking, cardiovascular risk factors, and chronic diseases as well as education level. Of note, interaction of age with household income was statistically significant, suggesting that the association of income with frailty is not modified by age. Our results suggested that the association with frailty is stronger among middle-aged than older adults. We found no independent association with education. Previous results on the associations of education with frailty have been inconsistent [16, 17].

Previous data on SES and frailty have been collected among people aged 65 years and over. Using a dataset from a health survey of 4,000 people aged 65 years and over living in all regions of Hong Kong, Woo et al. reported associations of geographic districts and socioeconomic status with frailty [18]; those of low SES had a higher risk of frailty. In the Cardiovascular Health Study (CHS) Hirsch et al. found that SES was not associated with frailty among African Americans [19]. Yet a previous report of the CHS that included Caucasian and African American participants aged 65 or more found that those who were frail had less education and lower income [2]. Both income and education were independently associated with incident frailty among the Women's Health Initiative study participants aged 65 to 79 [20]. In a cross-sectional analysis of the Women's Health and Aging studies (women aged 65 years or more), low education and low income were associated with frailty [8]. In an old adults (70+ yrs) Mexican population, having a poor self-perceived economic situation was more common in frail participants [21]. In community-dwelling individuals aged 65 and older in England, less wealth and greater neighborhood deprivation were associated with greater frailty [22]. Finally, in a small (*n* = 640) cross-sectional study of community-dwelling persons aged 75 and older in Lleida, Spain, education was not associated with frailty, while the association of frailty with income approached statistical significance [23]. We found that the association of income with frailty persisted after adjustment for comorbidity. This observation challenges the suggestion that social inequalities in frailty are mediated by comorbidity [10].

The association of SES and income with frailty is of particular importance in a Swiss region with universal healthcare coverage. Theou et al. recently reported that country level of frailty is correlated with national economic indicators. Our results highlight the fact that, even in a high-income country such as Switzerland, frailty is associated with income [24].

Chronic diseases have been associated with frailty [25]. In our study, only hypertension was statistically significantly associated with the number of frailty indicators. This is in line with previous studies conducted among older adults [2, 12]. Diabetes, myocardial infarction, angina pectoris, and arterial obstruction were not associated. Compared to these factors, hypertension was much more prevalent in our study population and it is therefore possible that we lacked power to detect associations for these less frequent factors.

Both BMI and waist-to-hip ratio were negatively associated with the number of frailty indicators. Frailty is conceptualized as a wasting disorder and sarcopenia is one of frailty major pathophysiological features. Therefore our results are concordant with the frailty syndrome. Yet, some previous studies found BMI and waist circumference to be positively associated with frailty [11, 26]. This relationship remains to be better determined.

We found an inverse relationship between total cholesterol and frailty. The relationships between lipid profiles and frailty have rarely been explored [11]. In a study on 637 hospitalized old patients, Ranieri et al. concluded that lower serum cholesterol levels were an independent hematologic marker of frailty [27]. Low cholesterol level is a marker of malnutrition and could therefore be noncausally associated with frailty status. More recently, Schupf et al. also concluded that low cholesterol level is a robust predictor of mortality in nondemented old adults and may be a surrogate of frailty or subclinical disease [28].

When interpreting the findings of this study, one has to keep in mind its limitations. While four of the five frailty indicators were collected using standard procedures, information on walking speed was missing and conventional definition of frailty syndrome (including prefrail and frail status) could not be used. Also using the conventional definition of frailty syndrome, having only one frailty indicator could perhaps predict who would be frail in the future but does not identify them as frail. All participants were assumed to have normal walking speed and the prevalence of frailty is therefore likely to be actually underestimated. Grip strength test was performed on the right hand. While, for left-handed persons, grip strength is considered equivalent in both hands, for right-handed persons the dominant hand possesses 10% greater grip strength than the nondominant hand [29]. Although significant efforts are made to avoid selection bias (e.g., random sampling, multiple means to contact potential participants, multiple recruitment sites including outside the hospital using a mobile examination unit, and no financial incentive), we cannot exclude that participants differ from nonparticipants with respect to some attributes that may potentially influence the presence or absence of frailty indicators. Several participant characteristics were determined by the use of questionnaires. By nature of its reliance on self-reported data, this is a source of possible bias. Due to the nature of information collected in the “Bus Santé” study (that collects information on cardiovascular risk factors in the Canton of Geneva), only diabetes and cardiovascular risk factors could be considered and not other health conditions potentially associated with frailty, such as other respiratory illnesses, arthritis, neurological disorders, depression/mental health, and cognitive impairment. Given the representativeness of the study sample, these conditions are likely to be prevalent and may therefore confound some of the associations reported. Also, we lack information on education level, which may play an important role in frailty, and cannot exclude that some of the associations reported are explained—at least in part—by education level.

## 5. Conclusions

In a Swiss region with universal healthcare coverage, frailty indicators are frequently encountered in both older and middle-aged adults from the general population. Frailty is partly reversible in its initial phase. This study identified determinants of frailty that can help in identifying vulnerable adults who could benefit from interventions to prevent adverse health outcomes and disability.

## Figures and Tables

**Table 1:** Participants' characteristics overall and by type of frailty indicator present, Bus Santé study, Geneva 2006–2010, *N* = 2,930.

Participant characteristic	All *N* = 2930 100%	Weakness *N* = 586 13.8%	Exhaustion *N* = 197 7.0%	Shrinking *N* = 188 6.3%	Low activity *N* = 586 18.3%
Age, years, mean (SE)	60.0 (0.1)	72.8 (0.1)	58.5 (0.5)	60.9 (0.6)	63.4 (0.3)
Age 50–65 years (%)	73.6	0.0	7.6	5.7	14.4
Age ≥ 65 years (%)	26.4	52.2	5.4	7.7	29.0
Women (%)	51.0	54.2	66.1	61.5	53.6
Swiss nationality (%)	78.8	79.4	76.9	74.7	79.0
Education level (%)					
Low	15.6	23.9	16.8	25.7	17.8
Middle	45.5	47.4	41.8	44.1	46.0
High	38.9	28.7	41.4	30.3	36.2
Household income (%)					
<4999	22.7	47.6	35.7	35.8	28.5
5000–9499	41.9	38.8	32.4	36.6	38.8
>9500	35.4	13.7	31.9	27.6	32.6
Hypertension (%)	47.5	70.2	53.4	49.9	53.1
Diabetes (%)	6.6	9.1	8.6	8.1	7.3
Myocardial infarction (%)	2.4	4.9	2.0	3.2	2.5
Angina pectoris (%)	2.7	4.9	5.4	4.4	2.9
Arterial obstruction (%)	2.9	5.6	8.1	4.6	2.7
Current smoker (%)	15.9	9.8	19.5	27.9	17.4
Body mass index, kg/m^2^, mean (SE)	26.1 (0.2)	26.4 (0.2)	28.0 (1.5)	24.9 (0.4)	24.7 (0.6)
Waist-to-hip ratio, mean (SE)	0.88 (0.00)	0.90 (0.00)	0.87 (0.01)	0.86 (0.01)	0.87 (0.01)
Serum total cholesterol, mmol/L, mean (SE)	5.63 (0.02)	5.58 (0.04)	5.59 (0.07)	5.51 (0.07)	5.60 (0.04)
Serum HDL cholesterol, mmol/L, mean (SE)	1.45 (0.01)	1.47 (0.02)	1.43 (0.03)	1.48 (0.03)	1.50 (0.02)
Serum triglycerides, mmol/L, mean (SE)	1.31 (0.02)	1.24 (0.02)	1.43 (0.08)	1.30 (0.07)	1.27 (0.03)

**Table 2:** Participants' characteristics by the number of frailty indicators present, Bus Santé study, Geneva, 2006–2010, *N* = 2,930.

Participant characteristic	0 frailty indicator *N* = 1711 63.5%	1 frailty indicator *N* = 921 28.7%	2 or more frailty indicators *N* = 298 7.8%	*P* value∗
	% or mean (SE)	% or mean (SE)	% or mean (SE)	
Age, years, mean (SE)	57.5 (0.1)	63.0 (0.3)	68.8 (0.5)	<0.0001
Women (%)	47.8	56.1	57.8	<0.0001
Age 50–65 years (%)	87.1	57.2	24.7	<0.0001
Age ≥ 65 years (%)	12.9	42.8	75.3	<0.0001
Swiss nationality (%)	78.5	80.3	75.1	0.183
Education level (%)				
Low	13.4	18.0	24.4	<0.0001
Middle	44.9	47.6	42.4	
High	41.6	34.4	33.2	
Household income (%)				
<4999	16.6	28.8	49.0	<0.0001
5000–9499	43.5	41.5	31.0	
>9500	39.9	29.7	20.1	
Hypertension (%)	42.5	53.6	64.9	<0.0001
Diabetes (%)	5.8	7.5	8.8	0.069
Myocardial infarction (%)	2.1	2.9	3.6	0.170
Angina pectoris (%)	2.1	3.2	5.1	0.009
Cerebral or legs vascular obstruction (%)	2.2	3.7	5.6	0.001
Current smoker (%)	15.4	16.9	16.6	0.569
Body mass index, kg/m^2^, mean (SE)	26.3 (0.2)	25.7 (0.2)	26.0 (1.3)	0.076
Waist-to-hip ratio, mean (SE)	0.89 (0.00)	0.88 (0.00)	0.88 (0.01)	0.326
Serum total cholesterol, mmol/L, mean (SE)	5.64 (0.02)	5.65 (0.03)	5.48 (0.06)	0.027
Serum HDL cholesterol, mmol/L, mean (SE)	1.44 (0.01)	1.46 (0.01)	1.50 (0.02)	0.045
Serum triglycerides, mmol/L, mean (SE)	1.31 (0.02)	1.34 (0.03)	1.20 (0.03)	0.009

^*^Rao-Scott Chi-square test for dichotomous and categorical variables and *F* tests for continuous variables.

**Table 3:** Multivariate associations (odds ratios OR, 95% CI) of characteristics with groups of number of frailty indicators, Bus Santé study, Geneva, 2006–2010, *N* = 2,930.

Participant characteristic	Frailty indicators group (ref = 0)	OR (95% CI)
Age ≥ 65 years versus 50–65 years	2+ versus 0	19.4 (13.5–27.9)∗
	1 versus 0	4.9 (4.0–6.0)∗
Male	2+ versus 0	1.02 (0.66–1.59)
	1 versus 0	0.72 (0.55–0.94)∗
Swiss nationality	2+ versus 0	0.77 (0.54–1.11)
	1 versus 0	1.05 (0.83–1.32)
Education		
Low	2+ versus 0	1.17 (0.75–1.83)
	1 versus 0	1.28 (0.96–1.71)
Middle	2+ versus 0	0.82 (0.58–1.16)
	1 versus 0	1.09 (0.89–1.35)
High	2+ versus 0	Ref
	1 versus 0	Ref
Household income		
<4999 CHF	2+ versus 0	2.16 (1.36–3.44)^∗†^
	1 versus 0	1.31 (1.00–1.72)∗
5000–9499 CHF	2+ versus 0	0.83 (0.55–1.27)
	1 versus 0	0.94 (0.75–1.17)
>9500 CHF	2+ versus 0	Ref
	1 versus 0	Ref
Hypertension	2+ versus 0	1.88 (1.32–2.68)∗
	1 versus 0	1.40 (1.15–1.70)∗
Diabetes	2+ versus 0	1.19 (0.66–2.15)
	1 versus 0	1.20 (0.83–1.72)
Myocardial infarction	2+ versus 0	0.78 (0.37–1.64)
	1 versus 0	0.96 (0.54–1.71)
Angina pectoris	2+ versus 0	1.69 (0.83–3.43)
	1 versus 0	1.24 (0.73–2.10)
Cerebral or legs vascular obstruction	2+ versus 0	1.70 (0.85–3.41)^†^
	1 versus 0	1.40 (0.81–2.44)
Current smoker	2+ versus 0	1.83 (1.19–2.81)∗
	1 versus 0	1.43 (1.03–1.68)∗
Body mass index, kg/m^2^	2+ versus 0	0.99 (0.89–1.10)
	1 versus 0	0.96 (0.93–0.99)∗
Waist-to-hip ratio	2+ versus 0	0.02 (0.01–0.40)∗
	1 versus 0	0.73 (0.15–3.68)
Serum total cholesterol, mmol/L	2+ versus 0	0.82 (0.69–0.98)∗
	1 versus 0	0.98 (0.89–1.09)
Serum triglycerides, mmol/L	2+ versus 0	0.98 (0.76–1.27)
	1 versus 0	1.12 (0.99–1.26)
Serum HDL cholesterol, mmol/L	2+ versus 0	1.57 (0.95–2.59)
	1 versus 0	1.09 (0.82–1.47)

All models are adjusted for survey year, age, gender, Swiss nationality, education level, monthly household income, hypertension, diabetes, myocardial infarction, angina pectoris, arterial obstruction, current smoking status, BMI, waist-to-hip ratio, serum total cholesterol, serum triglycerides, and serum HDL cholesterol.

∗Statistically significant associations (*P* value < 0.05); ^†^statistically significant interactions (*P* value < 0.05) with age category (50–64.9 years versus 65 years or more).

(1 CHF ≈ 1 US$.)
